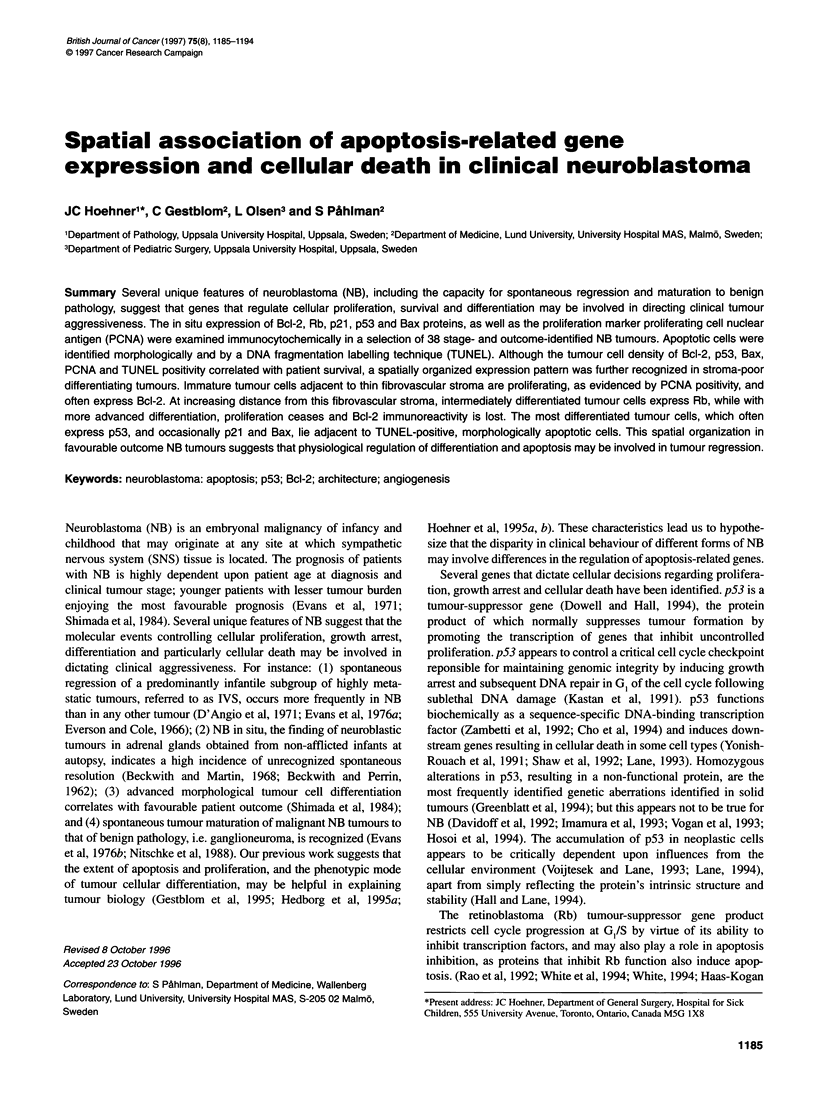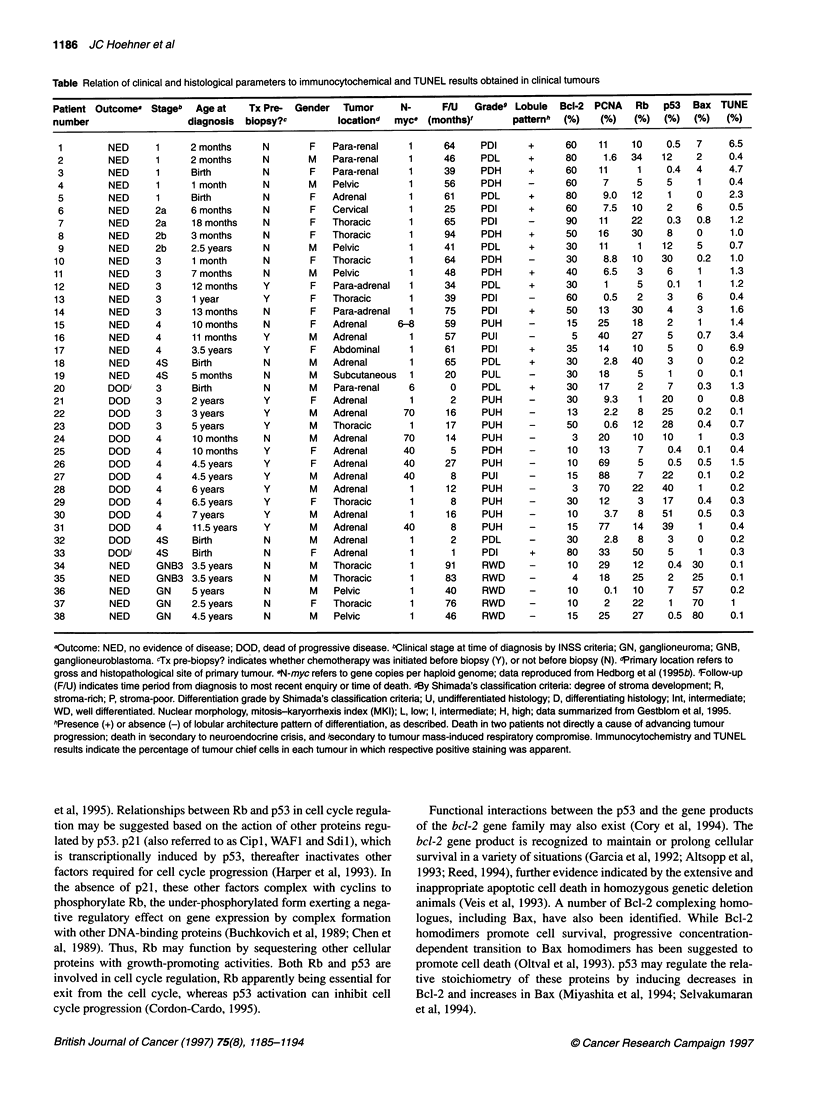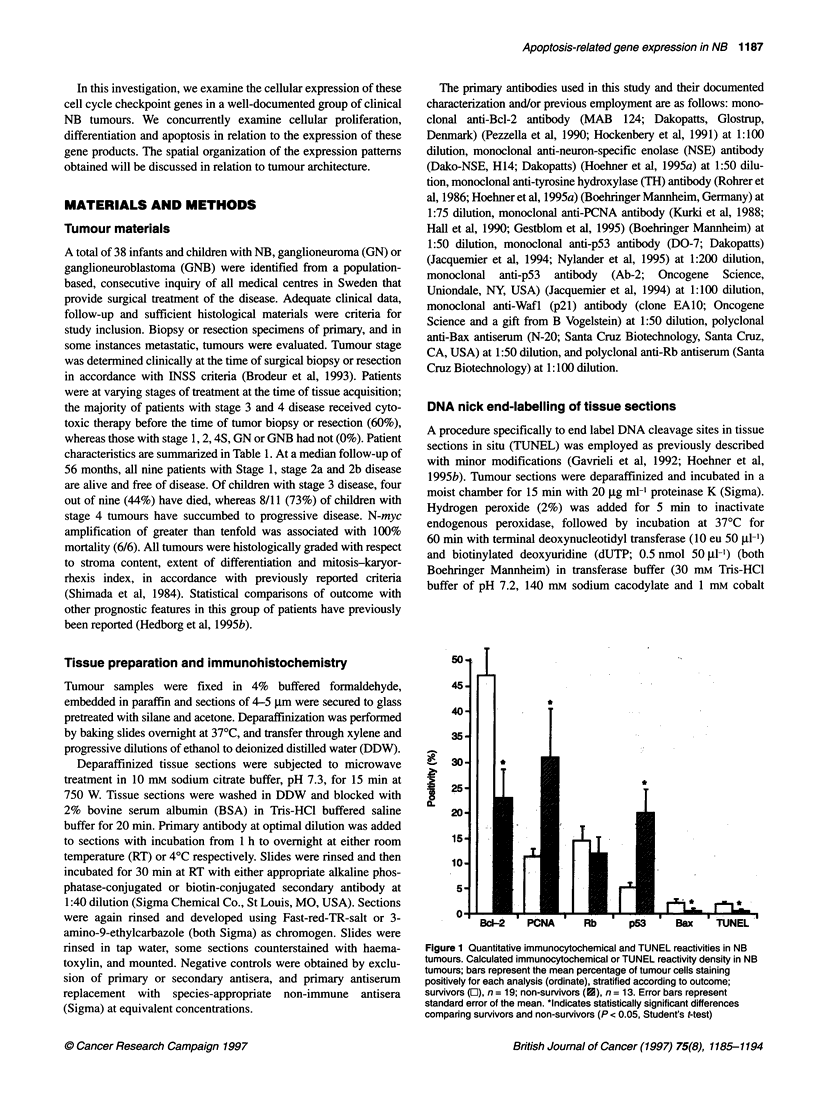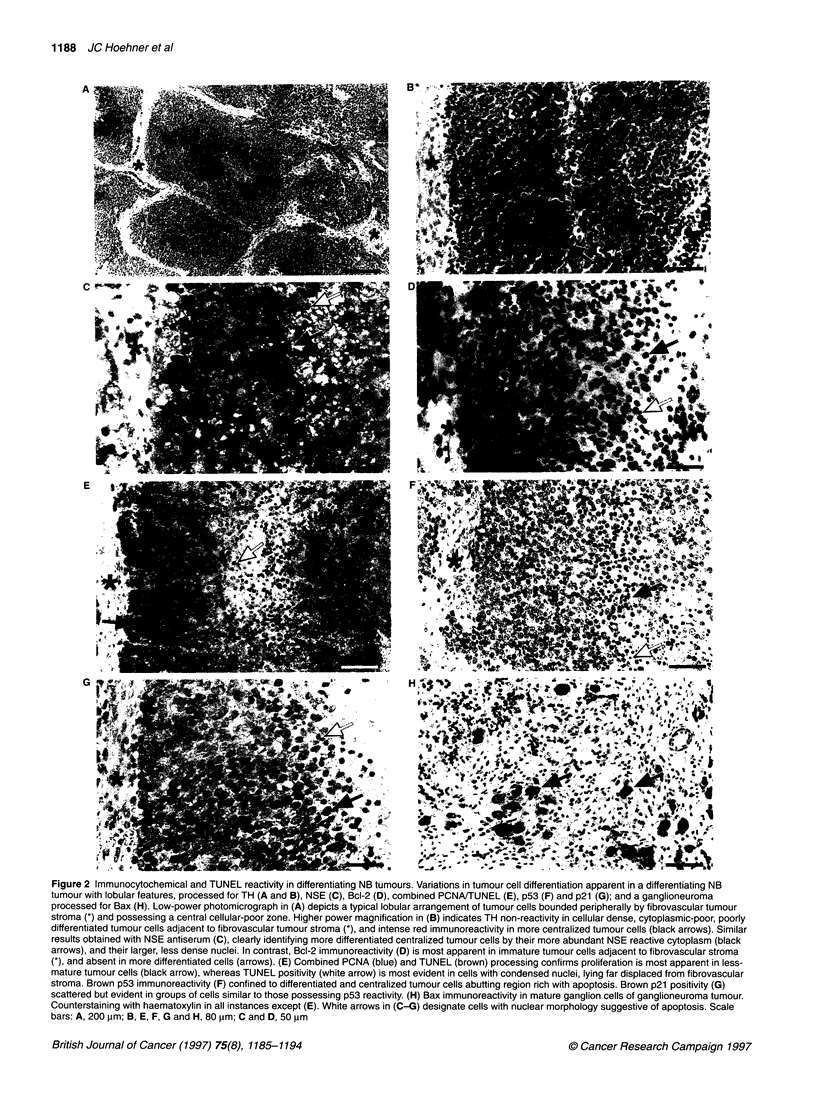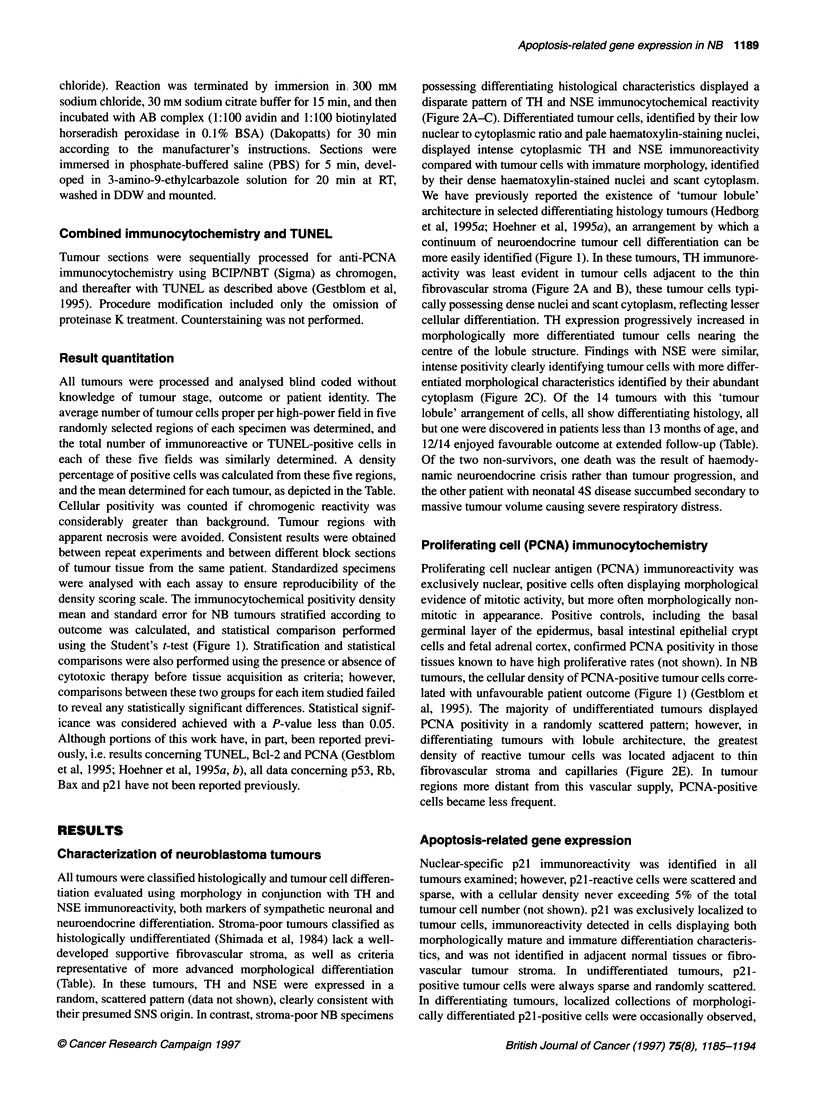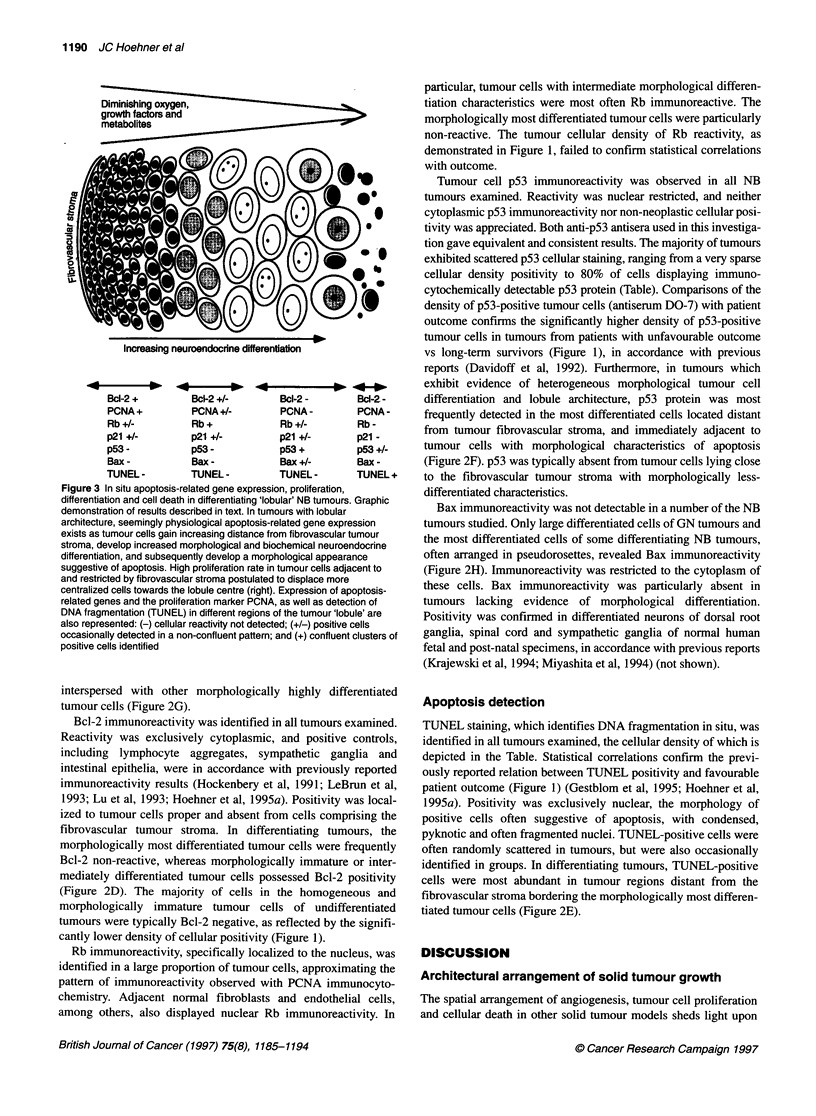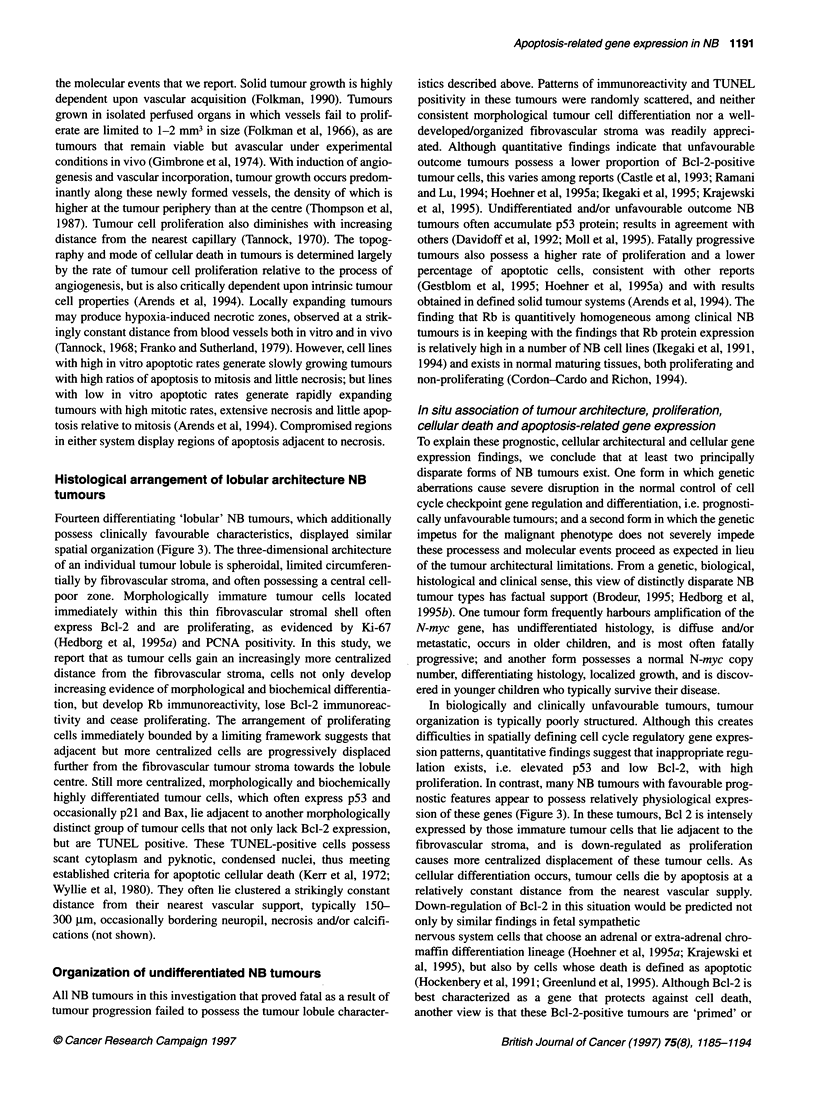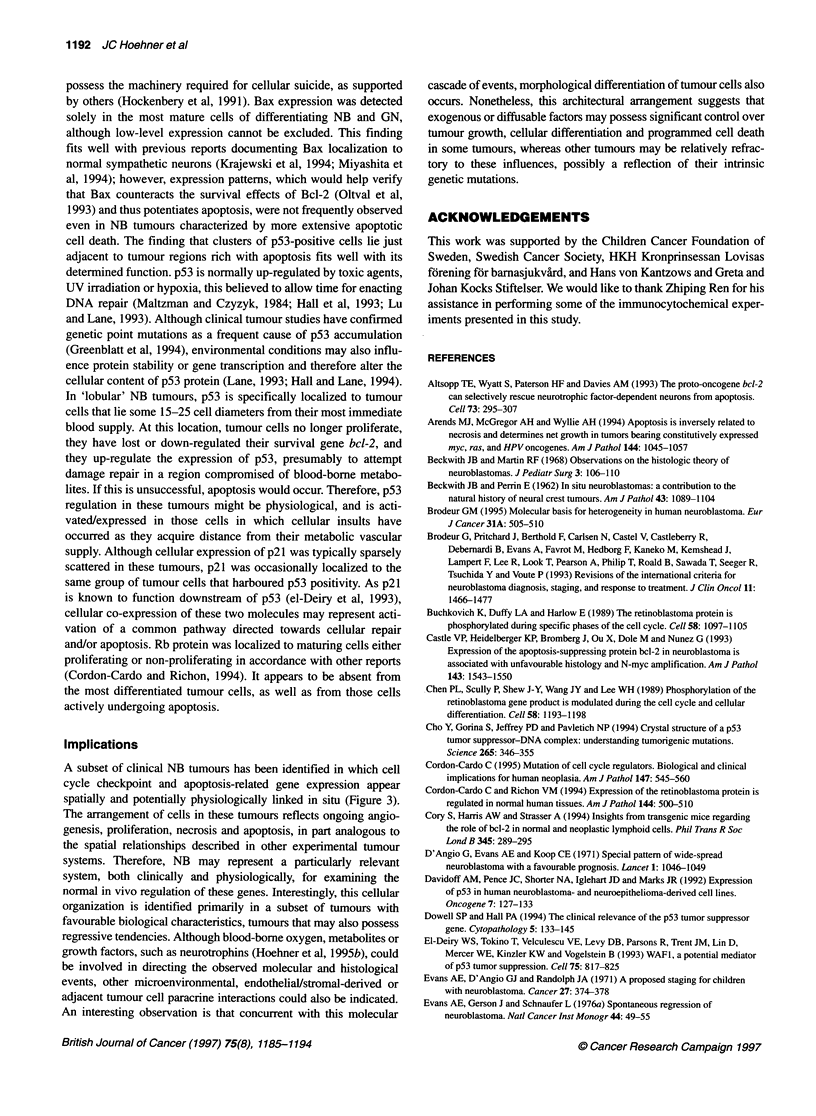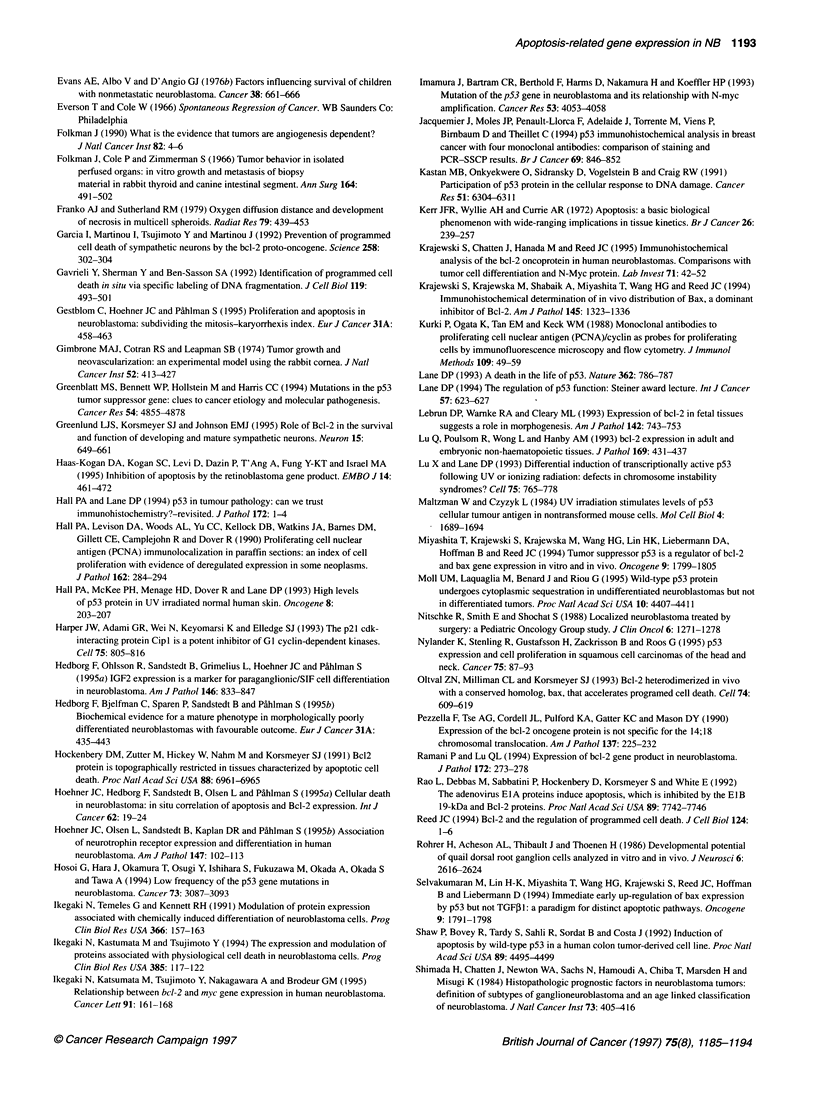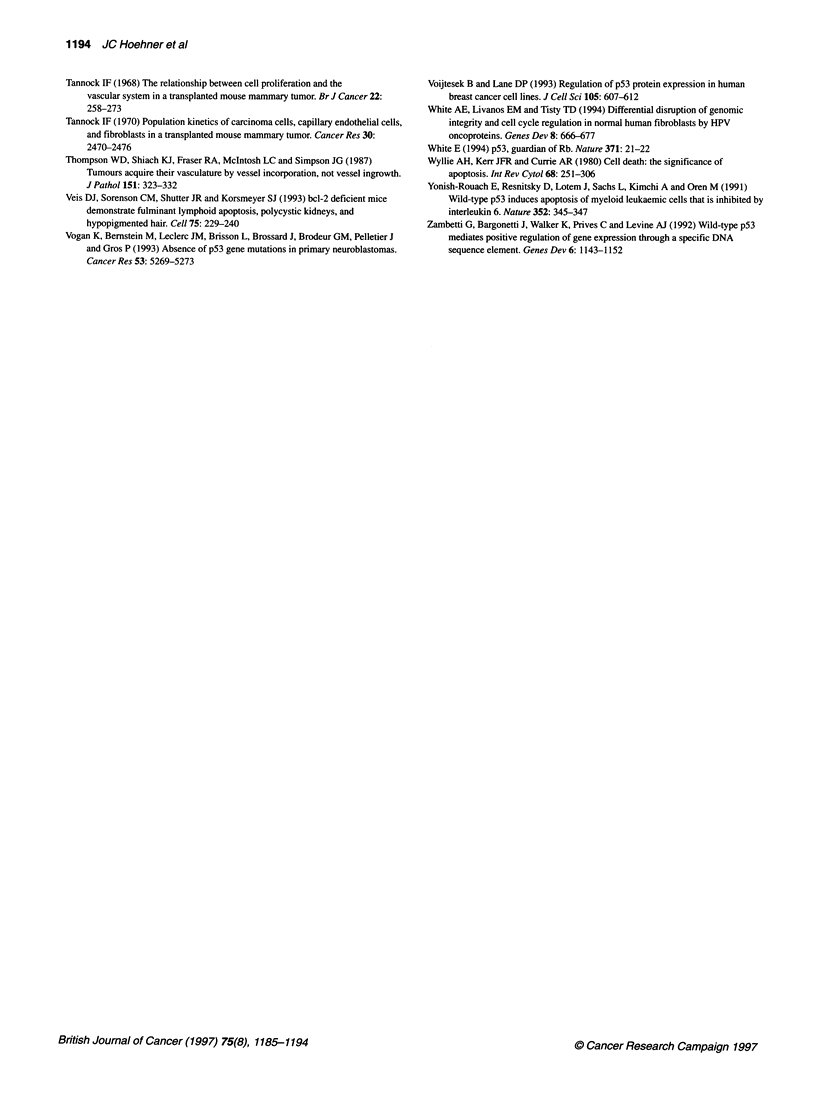# Spatial association of apoptosis-related gene expression and cellular death in clinical neuroblastoma.

**DOI:** 10.1038/bjc.1997.203

**Published:** 1997

**Authors:** J. C. Hoehner, C. Gestblom, L. Olsen, S. PÃ¥hlman

**Affiliations:** Department of Pathology, Uppsala University Hospital, Sweden.

## Abstract

**Images:**


British Journal of Cancer (1997) 75(8), 1185–1194
© 1997 Cancer Research Campaign

### Spatial association of apoptosis-related gene expression and cellular death in clinical neuroblastoma

JC Hoehner[1]*, C Gestblom[2], L Olsen[3] and S Påhlman[2]

[1]Department of Pathology, Uppsala University Hospital, Uppsala, Sweden; [2]Department of Medicine, Lund University, University Hospital MAS, Malmö, Sweden; [3]Department of Pediatric Surgery, Uppsala University Hospital, Uppsala, Sweden

**Summary** Several unique features of neuroblastoma (NB), including the capacity for spontaneous regression and maturation to benign pathology, suggest that genes that regulate cellular proliferation, survival and differentiation may be involved in directing clinical tumour aggressiveness. The in situ expression of Bcl-2, Rb, p21, p53 and Bax proteins, as well as the proliferation marker proliferating cell nuclear antigen (PCNA) were examined immunocytochemically in a selection of 38 stage- and outcome-identified NB tumours. Apoptotic cells were identified morphologically and by a DNA fragmentation labelling technique (TUNEL). Although the tumour cell density of Bcl-2, p53, Bax, PCNA and TUNEL positivity correlated with patient survival, a spatially organized expression pattern was further recognized in stroma-poor differentiating tumours. Immature tumour cells adjacent to thin fibrovascular stroma are proliferating, as evidenced by PCNA positivity, and often express Bcl-2. At increasing distance from this fibrovascular stroma, intermediately differentiated tumour cells express Rb, while with more advanced differentiation, proliferation ceases and Bcl-2 immunoreactivity is lost. The most differentiated tumour cells, which often express p53, and occasionally p21 and Bax, lie adjacent to TUNEL-positive, morphologically apoptotic cells. This spatial organization in favourable outcome NB tumours suggests that physiological regulation of differentiation and apoptosis may be involved in tumour regression.

**Keywords:** neuroblastoma: apoptosis; p53; Bcl-2; architecture; angiogenesis

Neuroblastoma (NB) is an embryonal malignancy of infancy and childhood that may originate at any site at which sympathetic nervous system (SNS) tissue is located. The prognosis of patients with NB is highly dependent upon patient age at diagnosis and clinical tumour stage; younger patients with lesser tumour burden enjoying the most favourable prognosis (Evans et al, 1971; Shimada et al, 1984). Several unique features of NB suggest that the molecular events controlling cellular proliferation, growth arrest, differentiation and particularly cellular death may be involved in dictating clinical aggressiveness. For instance: (1) spontaneous regression of a predominantly infantile subgroup of highly metastatic tumours, referred to as IVS, occurs more frequently in NB than in any other tumour (D'Angio et al, 1971; Evans et al, 1976*a*; Everson and Cole, 1966); (2) NB in situ, the finding of neuroblastic tumours in adrenal glands obtained from non-afflicted infants at autopsy, indicates a high incidence of unrecognized spontaneous resolution (Beckwith and Martin, 1968; Beckwith and Perrin, 1962); (3) advanced morphological tumour cell differentiation correlates with favourable patient outcome (Shimada et al, 1984); and (4) spontaneous tumour maturation of malignant NB tumours to that of benign pathology, i.e. ganglioneuroma, is recognized (Evans et al, 1976*b*; Nitschke et al, 1988). Our previous work suggests that the extent of apoptosis and proliferation, and the phenotypic mode of tumour cellular differentiation, may be helpful in explaining tumour biology (Gestblom et al, 1995; Hedborg et al, 1995*a*; Hoehner et al, 1995*a*, *b*). These characteristics lead us to hypothesize that the disparity in clinical behaviour of different forms of NB may involve differences in the regulation of apoptosis-related genes.

Several genes that dictate cellular decisions regarding proliferation, growth arrest and cellular death have been identified. *p53* is a tumour-suppressor gene (Dowell and Hall, 1994), the protein product of which normally suppresses tumour formation by promoting the transcription of genes that inhibit uncontrolled proliferation. *p53* appears to control a critical cell cycle checkpoint reponsible for maintaining genomic integrity by inducing growth arrest and subsequent DNA repair in $G_1$ of the cell cycle following sublethal DNA damage (Kastan et al, 1991). p53 functions biochemically as a sequence-specific DNA-binding transcription factor (Zambetti et al, 1992; Cho et al, 1994) and induces downstream genes resulting in cellular death in some cell types (Yonish-Rouach et al, 1991; Shaw et al, 1992; Lane, 1993). Homozygous alterations in p53, resulting in a non-functional protein, are the most frequently identified genetic aberrations identified in solid tumours (Greenblatt et al, 1994); but this appears not to be true for NB (Davidoff et al, 1992; Imamura et al, 1993; Vogan et al, 1993; Hosoi et al, 1994). The accumulation of p53 in neoplastic cells appears to be critically dependent upon influences from the cellular environment (Voijtesek and Lane, 1993; Lane, 1994), apart from simply reflecting the protein's intrinsic structure and stability (Hall and Lane, 1994).

The retinoblastoma (Rb) tumour-suppressor gene product restricts cell cycle progression at $G_1/S$ by virtue of its ability to inhibit transcription factors, and may also play a role in apoptosis inhibition, as proteins that inhibit Rb function also induce apoptosis. (Rao et al, 1992; White et al, 1994; White, 1994; Haas-Kogan

*Revised 8 October 1996*
*Accepted 23 October 1996*

*Correspondence to*: S Påhlman, Department of Medicine, Wallenberg Laboratory, Lund University, University Hospital MAS, S-205 02 Malmö, Sweden

*Present address: JC Hoehner, Department of General Surgery, Hospital for Sick Children, 555 University Avenue, Toronto, Ontario, Canada M5G 1X8

**Table** Relation of clinical and histological parameters to immunocytochemical and TUNEL results obtained in clinical tumours

| Patient number | Outcome[a] | Stage[b] | Age at diagnosis | Tx Pre-biopsy?[c] | Gender | Tumor location[d] | N-myc[e] | F/U (months)[f] | Grade[g] | Lobule pattern[h] | Bcl-2 (%) | PCNA (%) | Rb (%) | p53 (%) | Bax (%) | TUNE (%) |
|---|---|---|---|---|---|---|---|---|---|---|---|---|---|---|---|---|
| 1 | NED | 1 | 2 months | N | F | Para-renal | 1 | 64 | PDI | + | 60 | 11 | 10 | 0.5 | 7 | 6.5 |
| 2 | NED | 1 | 2 months | N | M | Para-renal | 1 | 46 | PDL | + | 80 | 1.6 | 34 | 12 | 2 | 0.4 |
| 3 | NED | 1 | Birth | N | F | Para-renal | 1 | 39 | PDH | + | 60 | 11 | 1 | 0.4 | 4 | 4.7 |
| 4 | NED | 1 | 1 month | N | M | Pelvic | 1 | 56 | PDH | – | 60 | 7 | 5 | 5 | 1 | 0.4 |
| 5 | NED | 1 | Birth | N | F | Adrenal | 1 | 61 | PDL | + | 80 | 9.0 | 12 | 1 | 0 | 2.3 |
| 6 | NED | 2a | 6 months | N | F | Cervical | 1 | 25 | PDI | + | 60 | 7.5 | 10 | 2 | 6 | 0.5 |
| 7 | NED | 2a | 18 months | N | F | Thoracic | 1 | 65 | PDI | – | 90 | 11 | 22 | 0.3 | 0.8 | 1.2 |
| 8 | NED | 2b | 3 months | N | F | Thoracic | 1 | 94 | PDH | + | 50 | 16 | 30 | 8 | 0 | 1.0 |
| 9 | NED | 2b | 2.5 years | N | M | Pelvic | 1 | 41 | PDL | + | 30 | 11 | 1 | 12 | 5 | 0.7 |
| 10 | NED | 3 | 1 month | N | F | Thoracic | 1 | 64 | PDH | – | 30 | 8.8 | 10 | 30 | 0.2 | 1.0 |
| 11 | NED | 3 | 7 months | N | M | Pelvic | 1 | 48 | PDH | + | 40 | 6.5 | 3 | 6 | 1 | 1.3 |
| 12 | NED | 3 | 12 months | Y | F | Para-adrenal | 1 | 34 | PDL | + | 30 | 1 | 5 | 0.1 | 1 | 1.2 |
| 13 | NED | 3 | 1 year | Y | F | Thoracic | 1 | 39 | PDI | – | 60 | 0.5 | 2 | 3 | 6 | 0.4 |
| 14 | NED | 3 | 13 months | N | F | Para-adrenal | 1 | 75 | PDI | + | 50 | 13 | 30 | 4 | 3 | 1.6 |
| 15 | NED | 4 | 10 months | N | F | Adrenal | 6–8 | 59 | PUH | – | 15 | 25 | 18 | 2 | 1 | 1.4 |
| 16 | NED | 4 | 11 months | Y | M | Adrenal | 1 | 57 | PUI | – | 5 | 40 | 27 | 5 | 0.7 | 3.4 |
| 17 | NED | 4 | 3.5 years | Y | F | Abdominal | 1 | 61 | PDI | + | 35 | 14 | 10 | 5 | 0 | 6.9 |
| 18 | NED | 4S | Birth | N | M | Adrenal | 1 | 65 | PDL | + | 30 | 2.8 | 40 | 3 | 0 | 0.2 |
| 19 | NED | 4S | 5 months | N | M | Subcutaneous | 1 | 20 | PUL | – | 30 | 18 | 5 | 1 | 0 | 0.1 |
| 20 | DOD[i] | 3 | Birth | N | M | Para-renal | 6 | 0 | PDL | + | 30 | 17 | 2 | 7 | 0.3 | 1.3 |
| 21 | DOD | 3 | 2 years | Y | F | Adrenal | 1 | 2 | PUH | – | 30 | 9.3 | 1 | 20 | 0 | 0.8 |
| 22 | DOD | 3 | 3 years | Y | M | Adrenal | 70 | 16 | PUH | – | 13 | 2.2 | 8 | 25 | 0.2 | 0.1 |
| 23 | DOD | 3 | 5 years | Y | M | Thoracic | 1 | 17 | PUH | – | 50 | 0.6 | 12 | 28 | 0.4 | 0.7 |
| 24 | DOD | 4 | 10 months | N | M | Adrenal | 70 | 14 | PUH | – | 3 | 20 | 10 | 10 | 1 | 0.3 |
| 25 | DOD | 4 | 10 months | Y | F | Adrenal | 40 | 5 | PDH | – | 10 | 13 | 7 | 0.4 | 0.1 | 0.4 |
| 26 | DOD | 4 | 4.5 years | Y | F | Adrenal | 40 | 27 | PUH | – | 10 | 69 | 5 | 0.5 | 0.5 | 1.5 |
| 27 | DOD | 4 | 4.5 years | Y | M | Adrenal | 40 | 8 | PUI | – | 15 | 88 | 7 | 22 | 0.1 | 0.2 |
| 28 | DOD | 4 | 6 years | Y | M | Adrenal | 1 | 12 | PUH | – | 3 | 70 | 22 | 40 | 1 | 0.2 |
| 29 | DOD | 4 | 6.5 years | Y | F | Thoracic | 1 | 8 | PUH | – | 30 | 12 | 3 | 17 | 0.4 | 0.3 |
| 30 | DOD | 4 | 7 years | Y | M | Adrenal | 1 | 16 | PUH | – | 10 | 3.7 | 8 | 51 | 0.5 | 0.3 |
| 31 | DOD | 4 | 11.5 years | Y | M | Adrenal | 40 | 8 | PUH | – | 15 | 77 | 14 | 39 | 1 | 0.4 |
| 32 | DOD | 4S | Birth | N | M | Adrenal | 1 | 2 | PDL | – | 30 | 2.8 | 8 | 3 | 0 | 0.2 |
| 33 | DOD[j] | 4S | Birth | N | F | Adrenal | 1 | 1 | PDI | + | 80 | 33 | 50 | 5 | 1 | 0.3 |
| 34 | NED | GNB3 | 3.5 years | N | M | Thoracic | 1 | 91 | RWD | – | 10 | 29 | 12 | 0.4 | 30 | 0.1 |
| 35 | NED | GNB3 | 3.5 years | N | M | Thoracic | 1 | 83 | RWD | – | 4 | 18 | 25 | 2 | 25 | 0.1 |
| 36 | NED | GN | 5 years | N | M | Pelvic | 1 | 40 | RWD | – | 10 | 0.1 | 10 | 7 | 57 | 0.2 |
| 37 | NED | GN | 2.5 years | N | F | Thoracic | 1 | 76 | RWD | – | 10 | 2 | 22 | 1 | 70 | 1 |
| 38 | NED | GN | 4.5 years | N | M | Pelvic | 1 | 46 | RWD | – | 15 | 25 | 27 | 0.5 | 80 | 0.1 |

[a]Outcome: NED, no evidence of disease; DOD, dead of progressive disease. [b]Clinical stage at time of diagnosis by INSS criteria; GN, ganglioneuroma; GNB, ganglioneuroblastoma. [c]Tx pre-biopsy? indicates whether chemotherapy was initiated before biopsy (Y), or not before biopsy (N). [d]Primary location refers to gross and histopathological site of primary tumour. [e]N-*myc* refers to gene copies per haploid genome; data reproduced from Hedborg et al (1995*b*). [f]Follow-up (F/U) indicates time period from diagnosis to most recent enquiry or time of death. [g]By Shimada's classification criteria: degree of stroma development; R, stroma-rich; P, stroma-poor. Differentiation grade by Shimada's classification criteria; U, undifferentiated histology; D, differentiating histology; Int, intermediate; WD, well differentiated. Nuclear morphology, mitosis–karyorrhexis index (MKI); L, low; I, intermediate; H, high; data summarized from Gestblom et al, 1995. [h]Presence (+) or absence (–) of lobular architecture pattern of differentiation, as described. Death in two patients not directly a cause of advancing tumour progression; death in [i]secondary to neuroendocrine crisis, and [j]secondary to tumour mass-induced respiratory compromise. Immunocytochemistry and TUNEL results indicate the percentage of tumour chief cells in each tumour in which respective positive staining was apparent.

et al, 1995). Relationships between Rb and p53 in cell cycle regulation may be suggested based on the action of other proteins regulated by p53. p21 (also referred to as Cip1, WAF1 and Sdi1), which is transcriptionally induced by p53, thereafter inactivates other factors required for cell cycle progression (Harper et al, 1993). In the absence of p21, these other factors complex with cyclins to phosphorylate Rb, the under-phosphorylated form exerting a negative regulatory effect on gene expression by complex formation with other DNA-binding proteins (Buchkovich et al, 1989; Chen et al, 1989). Thus, Rb may function by sequestering other cellular proteins with growth-promoting activities. Both Rb and p53 are involved in cell cycle regulation, Rb apparently being essential for exit from the cell cycle, whereas p53 activation can inhibit cell cycle progression (Cordon-Cardo, 1995).

Functional interactions between the p53 and the gene products of the *bcl-2* gene family may also exist (Cory et al, 1994). The *bcl-2* gene product is recognized to maintain or prolong cellular survival in a variety of situations (Garcia et al, 1992; Altsopp et al, 1993; Reed, 1994), further evidence indicated by the extensive and inappropriate apoptotic cell death in homozygous genetic deletion animals (Veis et al, 1993). A number of Bcl-2 complexing homologues, including Bax, have also been identified. While Bcl-2 homodimers promote cell survival, progressive concentration-dependent transition to Bax homodimers has been suggested to promote cell death (Oltval et al, 1993). p53 may regulate the relative stoichiometry of these proteins by inducing decreases in Bcl-2 and increases in Bax (Miyashita et al, 1994; Selvakumaran et al, 1994).

© Cancer Research Campaign 1997

In this investigation, we examine the cellular expression of these cell cycle checkpoint genes in a well-documented group of clinical NB tumours. We concurrently examine cellular proliferation, differentiation and apoptosis in relation to the expression of these gene products. The spatial organization of the expression patterns obtained will be discussed in relation to tumour architecture.

### MATERIALS AND METHODS

### Tumour materials

A total of 38 infants and children with NB, ganglioneuroma (GN) or ganglioneuroblastoma (GNB) were identified from a population-based, consecutive inquiry of all medical centres in Sweden that provide surgical treatment of the disease. Adequate clinical data, follow-up and sufficient histological materials were criteria for study inclusion. Biopsy or resection specimens of primary, and in some instances metastatic, tumours were evaluated. Tumour stage was determined clinically at the time of surgical biopsy or resection in accordance with INSS criteria (Brodeur et al, 1993). Patients were at varying stages of treatment at the time of tissue acquisition; the majority of patients with stage 3 and 4 disease received cytotoxic therapy before the time of tumor biopsy or resection (60%), whereas those with stage 1, 2, 4S, GN or GNB had not (0%). Patient characteristics are summarized in Table 1. At a median follow-up of 56 months, all nine patients with Stage 1, stage 2a and 2b disease are alive and free of disease. Of children with stage 3 disease, four out of nine (44%) have died, whereas 8/11 (73%) of children with stage 4 tumours have succumbed to progressive disease. N-*myc* amplification of greater than tenfold was associated with 100% mortality (6/6). All tumours were histologically graded with respect to stroma content, extent of differentiation and mitosis–karyorrhexis index, in accordance with previously reported criteria (Shimada et al, 1984). Statistical comparisons of outcome with other prognostic features in this group of patients have previously been reported (Hedborg et al, 1995*b*).

### Tissue preparation and immunohistochemistry

Tumour samples were fixed in 4% buffered formaldehyde, embedded in paraffin and sections of 4–5 μm were secured to glass pretreated with silane and acetone. Deparaffinization was performed by baking slides overnight at 37°C, and transfer through xylene and progressive dilutions of ethanol to deionized distilled water (DDW).

Deparaffinized tissue sections were subjected to microwave treatment in 10 mM sodium citrate buffer, pH 7.3, for 15 min at 750 W. Tissue sections were washed in DDW and blocked with 2% bovine serum albumin (BSA) in Tris-HCl buffered saline buffer for 20 min. Primary antibody at optimal dilution was added to sections with incubation from 1 h to overnight at either room temperature (RT) or 4°C respectively. Slides were rinsed and then incubated for 30 min at RT with either appropriate alkaline phosphatase-conjugated or biotin-conjugated secondary antibody at 1:40 dilution (Sigma Chemical Co., St Louis, MO, USA). Sections were again rinsed and developed using Fast-red-TR-salt or 3-amino-9-ethylcarbazole (both Sigma) as chromogen. Slides were rinsed in tap water, some sections counterstained with haematoxylin, and mounted. Negative controls were obtained by exclusion of primary or secondary antisera, and primary antiserum replacement with species-appropriate non-immune antisera (Sigma) at equivalent concentrations.

The primary antibodies used in this study and their documented characterization and/or previous employment are as follows: monoclonal anti-Bcl-2 antibody (MAB 124; Dakopatts, Glostrup, Denmark) (Pezzella et al, 1990; Hockenbery et al, 1991) at 1:100 dilution, monoclonal anti-neuron-specific enolase (NSE) antibody (Dako-NSE, H14; Dakopatts) (Hoehner et al, 1995*a*) at 1:50 dilution, monoclonal anti-tyrosine hydroxylase (TH) antibody (Rohrer et al, 1986; Hoehner et al, 1995*a*) (Boehringer Mannheim, Germany) at 1:75 dilution, monoclonal anti-PCNA antibody (Kurki et al, 1988; Hall et al, 1990; Gestblom et al, 1995) (Boehringer Mannheim) at 1:50 dilution, monoclonal anti-p53 antibody (DO-7; Dakopatts) (Jacquemier et al, 1994; Nylander et al, 1995) at 1:200 dilution, monoclonal anti-p53 antibody (Ab-2; Oncogene Science, Uniondale, NY, USA) (Jacquemier et al, 1994) at 1:100 dilution, monoclonal anti-Waf1 (p21) antibody (clone EA10; Oncogene Science and a gift from B Vogelstein) at 1:50 dilution, polyclonal anti-Bax antiserum (N-20; Santa Cruz Biotechnology, Santa Cruz, CA, USA) at 1:50 dilution, and polyclonal anti-Rb antiserum (Santa Cruz Biotechnology) at 1:100 dilution.

### DNA nick end-labelling of tissue sections

A procedure specifically to end label DNA cleavage sites in tissue sections in situ (TUNEL) was employed as previously described with minor modifications (Gavrieli et al, 1992; Hoehner et al, 1995*b*). Tumour sections were deparaffinized and incubated in a moist chamber for 15 min with 20 μg $ml^{-1}$ proteinase K (Sigma). Hydrogen peroxide (2%) was added for 5 min to inactivate endogenous peroxidase, followed by incubation at 37°C for 60 min with terminal deoxynucleotidyl transferase (10 eu 50 $\mu l^{-1}$) and biotinylated deoxyuridine (dUTP; 0.5 nmol 50 $\mu l^{-1}$) (both Boehringer Mannheim) in transferase buffer (30 mM Tris-HCl buffer of pH 7.2, 140 mM sodium cacodylate and 1 mM cobalt

**Figure 1** Quantitative immunocytochemical and TUNEL reactivities in NB tumours. Calculated immunocytochemical or TUNEL reactivity density in NB tumours; bars represent the mean percentage of tumour cells staining positively for each analysis (ordinate), stratified according to outcome; survivors (□), $n$ = 19; non-survivors (▨), $n$ = 13. Error bars represent standard error of the mean. *Indicates statistically significant differences comparing survivors and non-survivors ($P$ < 0.05, Student's $t$-test)

**Figure 2** Immunocytochemical and TUNEL reactivity in differentiating NB tumours. Variations in tumour cell differentiation apparent in a differentiating NB tumour with lobular features, processed for TH (**A** and **B**), NSE (**C**), Bcl-2 (**D**), combined PCNA/TUNEL (**E**), p53 (**F**) and p21 (**G**); and a ganglioneuroma processed for Bax (**H**). Low-power photomicrograph in (**A**) depicts a typical lobular arrangement of tumour cells bounded peripherally by fibrovascular tumour stroma (*) and possessing a central cellular-poor zone. Higher power magnification in (**B**) indicates TH non-reactivity in cellular dense, cytoplasmic-poor, poorly differentiated tumour cells adjacent to fibrovascular tumour stroma (*), and intense red immunoreactivity in more centralized tumour cells (black arrows). Similar results obtained with NSE antiserum (**C**), clearly identifying more differentiated centralized tumour cells by their more abundant NSE reactive cytoplasm (black arrows), and their larger, less dense nuclei. In contrast, Bcl-2 immunoreactivity (**D**) is most apparent in immature tumour cells adjacent to fibrovascular stroma (*), and absent in more differentiated cells (arrows). (**E**) Combined PCNA (blue) and TUNEL (brown) processing confirms proliferation is most apparent in less-mature tumour cells (black arrow), whereas TUNEL positivity (white arrow) is most evident in cells with condensed nuclei, lying far displaced from fibrovascular stroma. Brown p53 immunoreactivity (**F**) confined to differentiated and centralized tumour cells abutting region rich with apoptosis. Brown p21 positivity (**G**) scattered but evident in groups of cells similar to those possessing p53 reactivity. (**H**) Bax immunoreactivity in mature ganglion cells of ganglioneuroma tumour. Counterstaining with haematoxylin in all instances except (**E**). White arrows in (**C**–**G**) designate cells with nuclear morphology suggestive of apoptosis. Scale bars: **A**, 200 μm; **B**, **E**, **F**, **G** and **H**, 80 μm; **C** and **D**, 50 μm

© Cancer Research Campaign 1997

chloride). Reaction was terminated by immersion in 300 mM sodium chloride, 30 mM sodium citrate buffer for 15 min, and then incubated with AB complex (1:100 avidin and 1:100 biotinylated horseradish peroxidase in 0.1% BSA) (Dakopatts) for 30 min according to the manufacturer's instructions. Sections were immersed in phosphate-buffered saline (PBS) for 5 min, developed in 3-amino-9-ethylcarbazole solution for 20 min at RT, washed in DDW and mounted.

### Combined immunocytochemistry and TUNEL

Tumour sections were sequentially processed for anti-PCNA immunocytochemistry using BCIP/NBT (Sigma) as chromogen, and thereafter with TUNEL as described above (Gestblom et al, 1995). Procedure modification included only the omission of proteinase K treatment. Counterstaining was not performed.

### Result quantitation

All tumours were processed and analysed blind coded without knowledge of tumour stage, outcome or patient identity. The average number of tumour cells proper per high-power field in five randomly selected regions of each specimen was determined, and the total number of immunoreactive or TUNEL-positive cells in each of these five fields was similarly determined. A density percentage of positive cells was calculated from these five regions, and the mean determined for each tumour, as depicted in the Table. Cellular positivity was counted if chromogenic reactivity was considerably greater than background. Tumour regions with apparent necrosis were avoided. Consistent results were obtained between repeat experiments and between different block sections of tumour tissue from the same patient. Standardized specimens were analysed with each assay to ensure reproducibility of the density scoring scale. The immunocytochemical positivity density mean and standard error for NB tumours stratified according to outcome was calculated, and statistical comparison performed using the Student's *t*-test (Figure 1). Stratification and statistical comparisons were also performed using the presence or absence of cytotoxic therapy before tissue acquisition as criteria; however, comparisons between these two groups for each item studied failed to reveal any statistically significant differences. Statistical significance was considered achieved with a *P*-value less than 0.05. Although portions of this work have, in part, been reported previously, i.e. results concerning TUNEL, Bcl-2 and PCNA (Gestblom et al, 1995; Hoehner et al, 1995*a*, *b*), all data concerning p53, Rb, Bax and p21 have not been reported previously.

### RESULTS

### Characterization of neuroblastoma tumours

All tumours were classified histologically and tumour cell differentiation evaluated using morphology in conjunction with TH and NSE immunoreactivity, both markers of sympathetic neuronal and neuroendocrine differentiation. Stroma-poor tumours classified as histologically undifferentiated (Shimada et al, 1984) lack a well-developed supportive fibrovascular stroma, as well as criteria representative of more advanced morphological differentiation (Table). In these tumours, TH and NSE were expressed in a random, scattered pattern (data not shown), clearly consistent with their presumed SNS origin. In contrast, stroma-poor NB specimens possessing differentiating histological characteristics displayed a disparate pattern of TH and NSE immunocytochemical reactivity (Figure 2A–C). Differentiated tumour cells, identified by their low nuclear to cytoplasmic ratio and pale haematoxylin-staining nuclei, displayed intense cytoplasmic TH and NSE immunoreactivity compared with tumour cells with immature morphology, identified by their dense haematoxylin-stained nuclei and scant cytoplasm. We have previously reported the existence of 'tumour lobule' architecture in selected differentiating histology tumours (Hedborg et al, 1995*a*; Hoehner et al, 1995*a*), an arrangement by which a continuum of neuroendocrine tumour cell differentiation can be more easily identified (Figure 1). In these tumours, TH immunoreactivity was least evident in tumour cells adjacent to the thin fibrovascular stroma (Figure 2A and B), these tumour cells typically possessing dense nuclei and scant cytoplasm, reflecting lesser cellular differentiation. TH expression progressively increased in morphologically more differentiated tumour cells nearing the centre of the lobule structure. Findings with NSE were similar, intense positivity clearly identifying tumour cells with more differentiated morphological characteristics identified by their abundant cytoplasm (Figure 2C). Of the 14 tumours with this 'tumour lobule' arrangement of cells, all show differentiating histology, all but one were discovered in patients less than 13 months of age, and 12/14 enjoyed favourable outcome at extended follow-up (Table). Of the two non-survivors, one death was the result of haemodynamic neuroendocrine crisis rather than tumour progression, and the other patient with neonatal 4S disease succumbed secondary to massive tumour volume causing severe respiratory distress.

### Proliferating cell (PCNA) immunocytochemistry

Proliferating cell nuclear antigen (PCNA) immunoreactivity was exclusively nuclear, positive cells often displaying morphological evidence of mitotic activity, but more often morphologically non-mitotic in appearance. Positive controls, including the basal germinal layer of the epidermus, basal intestinal epithelial crypt cells and fetal adrenal cortex, confirmed PCNA positivity in those tissues known to have high proliferative rates (not shown). In NB tumours, the cellular density of PCNA-positive tumour cells correlated with unfavourable patient outcome (Figure 1) (Gestblom et al, 1995). The majority of undifferentiated tumours displayed PCNA positivity in a randomly scattered pattern; however, in differentiating tumours with lobule architecture, the greatest density of reactive tumour cells was located adjacent to thin fibrovascular stroma and capillaries (Figure 2E). In tumour regions more distant from this vascular supply, PCNA-positive cells became less frequent.

### Apoptosis-related gene expression

Nuclear-specific p21 immunoreactivity was identified in all tumours examined; however, p21-reactive cells were scattered and sparse, with a cellular density never exceeding 5% of the total tumour cell number (not shown). p21 was exclusively localized to tumour cells, immunoreactivity detected in cells displaying both morphologically mature and immature differentiation characteristics, and was not identified in adjacent normal tissues or fibrovascular tumour stroma. In undifferentiated tumours, p21-positive tumour cells were always sparse and randomly scattered. In differentiating tumours, localized collections of morphologically differentiated p21-positive cells were occasionally observed,

© Cancer Research Campaign 1997

**Figure 3** In situ apoptosis-related gene expression, proliferation, differentiation and cell death in differentiating 'lobular' NB tumours. Graphic demonstration of results described in text. In tumours with lobular architecture, seemingly physiological apoptosis-related gene expression exists as tumour cells gain increasing distance from fibrovascular tumour stroma, develop increased morphological and biochemical neuroendocrine differentiation, and subsequently develop a morphological appearance suggestive of apoptosis. High proliferation rate in tumour cells adjacent to and restricted by fibrovascular stroma postulated to displace more centralized cells towards the lobule centre (right). Expression of apoptosis-related genes and the proliferation marker PCNA, as well as detection of DNA fragmentation (TUNEL) in different regions of the tumour 'lobule' are also represented: (–) cellular reactivity not detected; (+/–) positive cells occasionally detected in a non-confluent pattern; and (+) confluent clusters of positive cells identified interspersed with other morphologically highly differentiated tumour cells (Figure 2G).

Bcl-2 immunoreactivity was identified in all tumours examined. Reactivity was exclusively cytoplasmic, and positive controls, including lymphocyte aggregates, sympathetic ganglia and intestinal epithelia, were in accordance with previously reported immunoreactivity results (Hockenbery et al, 1991; LeBrun et al, 1993; Lu et al, 1993; Hoehner et al, 1995*a*). Positivity was localized to tumour cells proper and absent from cells comprising the fibrovascular tumour stroma. In differentiating tumours, the morphologically most differentiated tumour cells were frequently Bcl-2 non-reactive, whereas morphologically immature or intermediately differentiated tumour cells possessed Bcl-2 positivity (Figure 2D). The majority of cells in the homogeneous and morphologically immature tumour cells of undifferentiated tumours were typically Bcl-2 negative, as reflected by the significantly lower density of cellular positivity (Figure 1).

Rb immunoreactivity, specifically localized to the nucleus, was identified in a large proportion of tumour cells, approximating the pattern of immunoreactivity observed with PCNA immunocytochemistry. Adjacent normal fibroblasts and endothelial cells, among others, also displayed nuclear Rb immunoreactivity. In particular, tumour cells with intermediate morphological differentiation characteristics were most often Rb immunoreactive. The morphologically most differentiated tumour cells were particularly non-reactive. The tumour cellular density of Rb reactivity, as demonstrated in Figure 1, failed to confirm statistical correlations with outcome.

Tumour cell p53 immunoreactivity was observed in all NB tumours examined. Reactivity was nuclear restricted, and neither cytoplasmic p53 immunoreactivity nor non-neoplastic cellular positivity was appreciated. Both anti-p53 antisera used in this investigation gave equivalent and consistent results. The majority of tumours exhibited scattered p53 cellular staining, ranging from a very sparse cellular density positivity to 80% of cells displaying immunocytochemically detectable p53 protein (Table). Comparisons of the density of p53-positive tumour cells (antiserum DO-7) with patient outcome confirms the significantly higher density of p53-positive tumour cells in tumours from patients with unfavourable outcome vs long-term survivors (Figure 1), in accordance with previous reports (Davidoff et al, 1992). Furthermore, in tumours which exhibit evidence of heterogeneous morphological tumour cell differentiation and lobule architecture, p53 protein was most frequently detected in the most differentiated cells located distant from tumour fibrovascular stroma, and immediately adjacent to tumour cells with morphological characteristics of apoptosis (Figure 2F). p53 was typically absent from tumour cells lying close to the fibrovascular tumour stroma with morphologically less-differentiated characteristics.

Bax immunoreactivity was not detectable in a number of the NB tumours studied. Only large differentiated cells of GN tumours and the most differentiated cells of some differentiating NB tumours, often arranged in pseudorosettes, revealed Bax immunoreactivity (Figure 2H). Immunoreactivity was restricted to the cytoplasm of these cells. Bax immunoreactivity was particularly absent in tumours lacking evidence of morphological differentiation. Positivity was confirmed in differentiated neurons of dorsal root ganglia, spinal cord and sympathetic ganglia of normal human fetal and post-natal specimens, in accordance with previous reports (Krajewski et al, 1994; Miyashita et al, 1994) (not shown).

### Apoptosis detection

TUNEL staining, which identifies DNA fragmentation in situ, was identified in all tumours examined, the cellular density of which is depicted in the Table. Statistical correlations confirm the previously reported relation between TUNEL positivity and favourable patient outcome (Figure 1) (Gestblom et al, 1995; Hoehner et al, 1995*a*). Positivity was exclusively nuclear, the morphology of positive cells often suggestive of apoptosis, with condensed, pyknotic and often fragmented nuclei. TUNEL-positive cells were often randomly scattered in tumours, but were also occasionally identified in groups. In differentiating tumours, TUNEL-positive cells were most abundant in tumour regions distant from the fibrovascular stroma bordering the morphologically most differentiated tumour cells (Figure 2E).

### DISCUSSION

### Architectural arrangement of solid tumour growth

The spatial arrangement of angiogenesis, tumour cell proliferation and cellular death in other solid tumour models sheds light upon

© Cancer Research Campaign 1997

the molecular events that we report. Solid tumour growth is highly dependent upon vascular acquisition (Folkman, 1990). Tumours grown in isolated perfused organs in which vessels fail to proliferate are limited to 1–2 $mm^3$ in size (Folkman et al, 1966), as are tumours that remain viable but avascular under experimental conditions in vivo (Gimbrone et al, 1974). With induction of angiogenesis and vascular incorporation, tumour growth occurs predominantly along these newly formed vessels, the density of which is higher at the tumour periphery than at the centre (Thompson et al, 1987). Tumour cell proliferation also diminishes with increasing distance from the nearest capillary (Tannock, 1970). The topography and mode of cellular death in tumours is determined largely by the rate of tumour cell proliferation relative to the process of angiogenesis, but is also critically dependent upon intrinsic tumour cell properties (Arends et al, 1994). Locally expanding tumours may produce hypoxia-induced necrotic zones, observed at a strikingly constant distance from blood vessels both in vitro and in vivo (Tannock, 1968; Franko and Sutherland, 1979). However, cell lines with high in vitro apoptotic rates generate slowly growing tumours with high ratios of apoptosis to mitosis and little necrosis; but lines with low in vitro apoptotic rates generate rapidly expanding tumours with high mitotic rates, extensive necrosis and little apoptosis relative to mitosis (Arends et al, 1994). Compromised regions in either system display regions of apoptosis adjacent to necrosis.

### Histological arrangement of lobular architecture NB tumours

Fourteen differentiating 'lobular' NB tumours, which additionally possess clinically favourable characteristics, displayed similar spatial organization (Figure 3). The three-dimensional architecture of an individual tumour lobule is spheroidal, limited circumferentially by fibrovascular stroma, and often possessing a central cell-poor zone. Morphologically immature tumour cells located immediately within this thin fibrovascular stromal shell often express Bcl-2 and are proliferating, as evidenced by Ki-67 (Hedborg et al, 1995*a*) and PCNA positivity. In this study, we report that as tumour cells gain an increasingly more centralized distance from the fibrovascular stroma, cells not only develop increasing evidence of morphological and biochemical differentiation, but develop Rb immunoreactivity, lose Bcl-2 immunoreactivity and cease proliferating. The arrangement of proliferating cells immediately bounded by a limiting framework suggests that adjacent but more centralized cells are progressively displaced further from the fibrovascular tumour stroma towards the lobule centre. Still more centralized, morphologically and biochemically highly differentiated tumour cells, which often express p53 and occasionally p21 and Bax, lie adjacent to another morphologically distinct group of tumour cells that not only lack Bcl-2 expression, but are TUNEL positive. These TUNEL-positive cells possess scant cytoplasm and pyknotic, condensed nuclei, thus meeting established criteria for apoptotic cellular death (Kerr et al, 1972; Wyllie et al, 1980). They often lie clustered a strikingly constant distance from their nearest vascular support, typically 150–300 μm, occasionally bordering neuropil, necrosis and/or calcifications (not shown).

### Organization of undifferentiated NB tumours

All NB tumours in this investigation that proved fatal as a result of tumour progression failed to possess the tumour lobule characteristics described above. Patterns of immunoreactivity and TUNEL positivity in these tumours were randomly scattered, and neither consistent morphological tumour cell differentiation nor a well-developed/organized fibrovascular stroma was readily appreciated. Although quantitative findings indicate that unfavourable outcome tumours possess a lower proportion of Bcl-2-positive tumour cells, this varies among reports (Castle et al, 1993; Ramani and Lu, 1994; Hoehner et al, 1995a; Ikegaki et al, 1995; Krajewski et al, 1995). Undifferentiated and/or unfavourable outcome NB tumours often accumulate p53 protein; results in agreement with others (Davidoff et al, 1992; Moll et al, 1995). Fatally progressive tumours also possess a higher rate of proliferation and a lower percentage of apoptotic cells, consistent with other reports (Gestblom et al, 1995; Hoehner et al, 1995a) and with results obtained in defined solid tumour systems (Arends et al, 1994). The finding that Rb is quantitively homogeneous among clinical NB tumours is in keeping with the findings that Rb protein expression is relatively high in a number of NB cell lines (Ikegaki et al, 1991, 1994) and exists in normal maturing tissues, both proliferating and non-proliferating (Cordon–Cardo and Richon, 1994).

### *In situ association of tumour architecture, proliferation, cellular death and apoptosis-related gene expression*

To explain these prognostic, cellular architectural and cellular gene expression findings, we conclude that at least two principally disparate forms of NB tumours exist. One form in which genetic aberrations cause severe disruption in the normal control of cell cycle checkpoint gene regulation and differentiation, i.e. prognostically unfavourable tumours; and a second form in which the genetic impetus for the malignant phenotype does not severely impede these processess and molecular events proceed as expected in lieu of the tumour architectural limitations. From a genetic, biological, histological and clinical sense, this view of distinctly disparate NB tumour types has factual support (Brodeur, 1995; Hedborg et al, 1995*b*). One tumour form frequently harbours amplification of the *N-myc* gene, has undifferentiated histology, is diffuse and/or metastatic, occurs in older children, and is most often fatally progressive; and another form possesses a normal N-*myc* copy number, differentiating histology, localized growth, and is discovered in younger children who typically survive their disease.

In biologically and clinically unfavourable tumours, tumour organization is typically poorly structured. Although this creates difficulties in spatially defining cell cycle regulatory gene expression patterns, quantitative findings suggest that inappropriate regulation exists, i.e. elevated p53 and low Bcl-2, with high proliferation. In contrast, many NB tumours with favourable prognostic features appear to possess relatively physiological expression of these genes (Figure 3). In these tumours, Bcl 2 is intensely expressed by those immature tumour cells that lie adjacent to the fibrovascular stroma, and is down-regulated as proliferation causes more centralized displacement of these tumour cells. As cellular differentiation occurs, tumour cells die by apoptosis at a relatively constant distance from the nearest vascular supply. Down-regulation of Bcl-2 in this situation would be predicted not only by similar findings in fetal sympathetic
nervous system cells that choose an adrenal or extra-adrenal chromaffin differentiation lineage (Hoehner et al, 1995*a*; Krajewski et al, 1995), but also by cells whose death is defined as apoptotic (Hockenbery et al, 1991; Greenlund et al, 1995). Although Bcl-2 is best characterized as a gene that protects against cell death, another view is that these Bcl-2-positive tumours are 'primed' or

© Cancer Research Campaign 1997

possess the machinery required for cellular suicide, as supported by others (Hockenbery et al, 1991). Bax expression was detected solely in the most mature cells of differentiating NB and GN, although low-level expression cannot be excluded. This finding fits well with previous reports documenting Bax localization to normal sympathetic neurons (Krajewski et al, 1994; Miyashita et al, 1994); however, expression patterns, which would help verify that Bax counteracts the survival effects of Bcl-2 (Oltval et al, 1993) and thus potentiates apoptosis, were not frequently observed even in NB tumours characterized by more extensive apoptotic cell death. The finding that clusters of p53-positive cells lie just adjacent to tumour regions rich with apoptosis fits well with its determined function. p53 is normally up-regulated by toxic agents, UV irradiation or hypoxia, this believed to allow time for enacting DNA repair (Maltzman and Czyzyk, 1984; Hall et al, 1993; Lu and Lane, 1993). Although clinical tumour studies have confirmed genetic point mutations as a frequent cause of p53 accumulation (Greenblatt et al, 1994), environmental conditions may also influence protein stability or gene transcription and therefore alter the cellular content of p53 protein (Lane, 1993; Hall and Lane, 1994). In 'lobular' NB tumours, p53 is specifically localized to tumour cells that lie some 15–25 cell diameters from their most immediate blood supply. At this location, tumour cells no longer proliferate, they have lost or down-regulated their survival gene *bcl-2*, and they up-regulate the expression of p53, presumably to attempt damage repair in a region compromised of blood-borne metabolites. If this is unsuccessful, apoptosis would occur. Therefore, p53 regulation in these tumours might be physiological, and is activated/expressed in those cells in which cellular insults have occurred as they acquire distance from their metabolic vascular supply. Although cellular expression of p21 was typically sparsely scattered in these tumours, p21 was occasionally localized to the same group of tumour cells that harboured p53 positivity. As p21 is known to function downstream of p53 (el-Deiry et al, 1993), cellular co-expression of these two molecules may represent activation of a common pathway directed towards cellular repair and/or apoptosis. Rb protein was localized to maturing cells either proliferating or non-proliferating in accordance with other reports (Cordon-Cardo and Richon, 1994). It appears to be absent from the most differentiated tumour cells, as well as from those cells actively undergoing apoptosis.

### Implications

A subset of clinical NB tumours has been identified in which cell cycle checkpoint and apoptosis-related gene expression appear spatially and potentially physiologically linked in situ (Figure 3). The arrangement of cells in these tumours reflects ongoing angiogenesis, proliferation, necrosis and apoptosis, in part analogous to the spatial relationships described in other experimental tumour systems. Therefore, NB may represent a particularly relevant system, both clinically and physiologically, for examining the normal in vivo regulation of these genes. Interestingly, this cellular organization is identified primarily in a subset of tumours with favourable biological characteristics, tumours that may also possess regressive tendencies. Although blood-borne oxygen, metabolites or growth factors, such as neurotrophins (Hoehner et al, 1995*b*), could be involved in directing the observed molecular and histological events, other microenvironmental, endothelial/stromal-derived or adjacent tumour cell paracrine interactions could also be indicated. An interesting observation is that concurrent with this molecular cascade of events, morphological differentiation of tumour cells also occurs. Nonetheless, this architectural arrangement suggests that exogenous or diffusable factors may possess significant control over tumour growth, cellular differentiation and programmed cell death in some tumours, whereas other tumours may be relatively refractory to these influences, possibly a reflection of their intrinsic genetic mutations.

### ACKNOWLEDGEMENTS

This work was supported by the Children Cancer Foundation of Sweden, Swedish Cancer Society, HKH Kronprinsessan Lovisas förening för barnasjukvård, and Hans von Kantzows and Greta and Johan Kocks Stiftelser. We would like to thank Zhiping Ren for his assistance in performing some of the immunocytochemical experiments presented in this study.

### REFERENCES

Altsopp TE, Wyatt S, Paterson HF and Davies AM (1993) The proto-oncogene *bcl-2* can selectively rescue neurotrophic factor-dependent neurons from apoptosis. *Cell* **73**: 295–307

Arends MJ, McGregor AH and Wyllie AH (1994) Apoptosis is inversely related to necrosis and determines net growth in tumors bearing constitutively expressed *myc*, *ras*, and *HPV* oncogenes. *Am J Pathol* **144**: 1045–1057

Beckwith JB and Martin RF (1968) Observations on the histologic theory of neuroblastomas. *J Pediatr Surg* **3**: 106–110

Beckwith JB and Perrin E (1962) In situ neuroblastomas: a contribution to the natural history of neural crest tumours. *Am J Pathol* **43**: 1089–1104

Brodeur GM (1995) Molecular basis for heterogeneity in human neuroblastoma. *Eur J Cancer* **31A**: 505–510

Brodeur G, Pritchard J, Berthold F, Carlsen N, Castel V, Castleberry R, Debernardi B, Evans A, Favrot M, Hedborg F, Kaneko M, Kemshead J, Lampert F, Lee R, Look T, Pearson A, Philip T, Roald B, Sawada T, Seeger R, Tsuchida Y and Voute P (1993) Revisions of the international criteria for neuroblastoma diagnosis, staging, and response to treatment. *J Clin Oncol* **11**: 1466–1477

Buchkovich K, Duffy LA and Harlow E (1989) The retinoblastoma protein is phosphorylated during specific phases of the cell cycle. *Cell* **58**: 1097–1105

Castle VP, Heidelberger KP, Bromberg J, Ou X, Dole M and Nunez G (1993) Expression of the apoptosis-suppressing protein bcl-2 in neuroblastoma is associated with unfavourable histology and N-myc amplification. *Am J Pathol* **143**: 1543–1550

Chen PL, Scully P, Shew J-Y, Wang JY and Lee WH (1989) Phosphorylation of the retinoblastoma gene product is modulated during the cell cycle and cellular differentiation. *Cell* **58**: 1193–1198

Cho Y, Gorina S, Jeffrey PD and Pavletich NP (1994) Crystal structure of a p53 tumor suppressor–DNA complex: understanding tumorigenic mutations. *Science* **265**: 346–355

Cordon-Cardo C (1995) Mutation of cell cycle regulators. Biological and clinical implications for human neoplasia. *Am J Pathol* **147**: 545–560

Cordon-Cardo C and Richon VM (1994) Expression of the retinoblastoma protein is regulated in normal human tissues. *Am J Pathol* **144**: 500–510

Cory S, Harris AW and Strasser A (1994) Insights from transgenic mice regarding the role of bcl-2 in normal and neoplastic lymphoid cells. *Phil Trans R Soc Lond B* **345**: 289–295

D'Angio G, Evans AE and Koop CE (1971) Special pattern of wide-spread neuroblastoma with a favourable prognosis. *Lancet* **1**: 1046–1049

Davidoff AM, Pence JC, Shorter NA, Iglehart JD and Marks JR (1992) Expression of p53 in human neuroblastoma- and neuroepithelioma-derived cell lines. *Oncogene* **7**: 127–133

Dowell SP and Hall PA (1994) The clinical relevance of the p53 tumor suppressor gene. *Cytopathology* **5**: 133–145

El-Deiry WS, Tokino T, Velculescu VE, Levy DB, Parsons R, Trent JM, Lin D, Mercer WE, Kinzler KW and Vogelstein B (1993) WAF1, a potential mediator of p53 tumor suppression. *Cell* **75**: 817–825

Evans AE, D'Angio GJ and Randolph JA (1971) A proposed staging for children with neuroblastoma. *Cancer* **27**: 374–378

Evans AE, Gerson J and Schnaufer L (1976*a*) Spontaneous regression of neuroblastoma. *Natl Cancer Inst Monogr* **44**: 49–55

*© Cancer Research Campaign 1997*

Evans AE, Albo V and D'Angio GJ (1976*b*) Factors influencing survival of children with nonmetastatic neuroblastoma. *Cancer* **38**: 661–666

Everson T and Cole W (1966) *Spontaneous Regression of Cancer*. WB Saunders Co: Philadelphia

Folkman J (1990) What is the evidence that tumors are angiogenesis dependent? *J Natl Cancer Inst* **82**: 4–6

Folkman J, Cole P and Zimmerman S (1966) Tumor behavior in isolated perfused organs: in vitro growth and metastasis of biopsy material in rabbit thyroid and canine intestinal segment. *Ann Surg* **164**: 491-502

Franko AJ and Sutherland RM (1979) Oxygen diffusion distance and development of necrosis in multicell spheroids. *Radiat Res* **79**: 439–453

Garcia I, Martinou I, Tsujimoto Y and Martinou J (1992) Prevention of programmed cell death of sympathetic neurons by the bcl-2 proto-oncogene. *Science* **258**: 302-304

Gavrieli Y, Sherman Y and Ben-Sasson SA (1992) Identification of programmed cell death *in situ* via specific labeling of DNA fragmentation. *J Cell Biol* **119**: 493-501

Gestblom C, Hoehner JC and Påhlman S (1995) Proliferation and apoptosis in neuroblastoma: subdividing the mitosis–karyorrhexis index. *Eur J Cancer* **31A**: 458–463

Gimbrone MAJ, Cotran RS and Leapman SB (1974) Tumor growth and neovascularization: an experimental model using the rabbit cornea. *J Natl Cancer Inst* **52**: 413–427

Greenblatt MS, Bennett WP, Hollstein M and Harris CC (1994) Mutations in the p53 tumor suppressor gene: clues to cancer etiology and molecular pathogenesis. *Cancer Res* **54**: 4855–4878

Greenlund LJS, Korsmeyer SJ and Johnson EMJ (1995) Role of Bcl-2 in the survival and function of developing and mature sympathetic neurons. *Neuron* **15**: 649-661

Haas-Kogan DA, Kogan SC, Levi D, Dazin P, T'Ang A, Fung Y-KT and Israel MA (1995) Inhibition of apoptosis by the retinoblastoma gene product. *EMBO J* **14**: 461–472

Hall PA and Lane DP (1994) p53 in tumour pathology: can we trust immunohistochemistry?–revisited. *J Pathol* **172**: 1–4

Hall PA, Levison DA, Woods AL, Yu CC, Kellock DB, Watkins JA, Barnes DM, Gillett CE, Camplejohn R and Dover R (1990) Proliferating cell nuclear antigen (PCNA) immunolocalization in paraffin sections: an index of cell proliferation with evidence of deregulated expression in some neoplasms. *J Pathol* **162**: 284–294

Hall PA, McKee PH, Menage HD, Dover R and Lane DP (1993) High levels of p53 protein in UV irradiated normal human skin. *Oncogene* **8**: 203-207

Harper JW, Adami GR, Wei N, Keyomarsi K and Elledge SJ (1993) The p21 cdk-interacting protein Cip1 is a potent inhibitor of G1 cyclin-dependent kinases. *Cell* **75**: 805-816

Hedborg F, Ohlsson R, Sandstedt B, Grimelius L, Hoehner JC and Påhlman S (1995*a*) IGF2 expression is a marker for paraganglionic/SIF cell differentiation in neuroblastoma. *Am J Pathol* **146**: 833–847

Hedborg F, Bjelfman C, Sparen P, Sandstedt B and Påhlman S (1995*b*) Biochemical evidence for a mature phenotype in morphologically poorly differentiated neuroblastomas with favourable outcome. *Eur J Cancer* **31A**: 435–443

Hockenbery DM, Zutter M, Hickey W, Nahm M and Korsmeyer SJ (1991) Bcl2 protein is topographically restricted in tissues characterized by apoptotic cell death. *Proc Natl Acad Sci USA* **88**: 6961–6965

Hoehner JC, Hedborg F, Sandstedt B, Olsen L and Påhlman S (1995*a*) Cellular death in neuroblastoma: in situ correlation of apoptosis and Bcl-2 expression. *Int J Cancer* **62**: 19–24

Hoehner JC, Olsen L, Sandstedt B, Kaplan DR and Påhlman S (1995*b*) Association of neurotrophin receptor expression and differentiation in human neuroblastoma. *Am J Pathol* **147**: 102–113

Hosoi G, Hara J, Okamura T, Osugi Y, Ishihara S, Fukuzawa M, Okada A, Okada S and Tawa A (1994) Low frequency of the p53 gene mutations in neuroblastoma. *Cancer* **73**: 3087–3093

Ikegaki N, Temeles G and Kennett RH (1991) Modulation of protein expression associated with chemically induced differentiation of neuroblastoma cells. *Prog Clin Biol Res USA* **366**: 157–163

Ikegaki N, Kastumata M and Tsujimoto Y (1994) The expression and modulation of proteins associated with physiological cell death in neuroblastoma cells. *Prog Clin Biol Res USA* **385**: 117–122

Ikegaki N, Katsumata M, Tsujimoto Y, Nakagawara A and Brodeur GM (1995) Relationship between *bcl-2* and *myc* gene expression in human neuroblastoma. *Cancer Lett* **91**: 161–168

Imamura J, Bartram CR, Berthold F, Harms D, Nakamura H and Koeffler HP (1993) Mutation of the *p53* gene in neuroblastoma and its relationship with N-myc amplification. *Cancer Res* **53**: 4053–4058

Jacquemier J, Moles JP, Penault-Llorca F, Adelaide J, Torrente M, Viens P, Birnbaum D and Theillet C (1994) p53 immunohistochemical analysis in breast cancer with four monoclonal antibodies: comparison of staining and PCR–SSCP results. *Br J Cancer* **69**: 846–852

Kastan MB, Onkyekwere O, Sidransky D, Vogelstein B and Craig RW (1991) Participation of p53 protein in the cellular response to DNA damage. *Cancer Res* **51**: 6304–6311

Kerr JFR, Wyllie AH and Currie AR (1972) Apoptosis: a basic biological phenomenon with wide-ranging implications in tissue kinetics. *Br J Cancer* **26**: 239–257

Krajewski S, Chatten J, Hanada M and Reed JC (1995) Immunohistochemical analysis of the bcl-2 oncoprotein in human neuroblastomas. Comparisons with tumor cell differentiation and N-Myc protein. *Lab Invest* **71**: 42–52

Krajewski S, Krajewska M, Shabaik A, Miyashita T, Wang HG and Reed JC (1994) Immunohistochemical determination of in vivo distribution of Bax, a dominant inhibitor of Bcl-2. *Am J Pathol* **145**: 1323–1336

Kurki P, Ogata K, Tan EM and Keck WM (1988) Monoclonal antibodies to proliferating cell nuclear antigen (PCNA)/cyclin as probes for proliferating cells by immunofluorescence microscopy and flow cytometry. *J Immunol Methods* **109**: 49–59

Lane DP (1993) A death in the life of p53. *Nature* **362**: 786–787

Lane DP (1994) The regulation of p53 function: Steiner award lecture. *Int J Cancer* **57**: 623–627

Lebrun DP, Warnke RA and Cleary ML (1993) Expression of bcl-2 in fetal tissues suggests a role in morphogenesis. *Am J Pathol* **142**: 743–753

Lu Q, Poulsom R, Wong L and Hanby AM (1993) bcl-2 expression in adult and embryonic non-haematopoietic tissues. *J Pathol* **169**: 431–437

Lu X and Lane DP (1993) Differential induction of transcriptionally active p53 following UV or ionizing radiation: defects in chromosome instability syndromes? *Cell* **75**: 765–778

Maltzman W and Czyzyk L (1984) UV irradiation stimulates levels of p53 cellular tumour antigen in nontransformed mouse cells. *Mol Cell Biol* **4**: 1689–1694

Miyashita T, Krajewski S, Krajewska M, Wang HG, Lin HK, Liebermann DA, Hoffman B and Reed JC (1994) Tumor suppressor p53 is a regulator of bcl-2 and bax gene expression in vitro and in vivo. *Oncogene* **9**: 1799–1805

Moll UM, Laquaglia M, Benard J and Riou G (1995) Wild-type p53 protein undergoes cytoplasmic sequestration in undifferentiated neuroblastomas but not in differentiated tumors. *Proc Natl Acad Sci USA* **10**: 4407–4411

Nitschke R, Smith E and Shochat S (1988) Localized neuroblastoma treated by surgery: a Pediatric Oncology Group study. *J Clin Oncol* **6**: 1271–1278

Nylander K, Stenling R, Gustafsson H, Zackrisson B and Roos G (1995) p53 expression and cell proliferation in squamous cell carcinomas of the head and neck. *Cancer* **75**: 87–93

Oltval ZN, Milliman CL and Korsmeyer SJ (1993) Bcl-2 heterodimerized in vivo with a conserved homolog, bax, that accelerates programed cell death. *Cell* **74**: 609–619

Pezzella F, Tse AG, Cordell JL, Pulford KA, Gatter KC and Mason DY (1990) Expression of the bcl-2 oncogene protein is not specific for the 14;18 chromosomal translocation. *Am J Pathol* **137**: 225–232

Ramani P and Lu QL (1994) Expression of bcl-2 gene product in neuroblastoma. *J Pathol* **172**: 273–278

Rao L, Debbas M, Sabbatini P, Hockenbery D, Korsmeyer S and White E (1992) The adenovirus E1A proteins induce apoptosis, which is inhibited by the E1B 19-kDa and Bcl-2 proteins. *Proc Natl Acad Sci USA* **89**: 7742–7746

Reed JC (1994) Bcl-2 and the regulation of programmed cell death. *J Cell Biol* **124**: 1–6

Rohrer H, Acheson AL, Thibault J and Thoenen H (1986) Developmental potential of quail dorsal root ganglion cells analyzed in vitro and in vivo. *J Neurosci* **6**: 2616–2624

Selvakumaran M, Lin H-K, Miyashita T, Wang HG, Krajewski S, Reed JC, Hoffman B and Liebermann D (1994) Immediate early up-regulation of bax expression by p53 but not TGFβ1: a paradigm for distinct apoptotic pathways. *Oncogene* **9**: 1791–1798

Shaw P, Bovey R, Tardy S, Sahli R, Sordat B and Costa J (1992) Induction of apoptosis by wild-type p53 in a human colon tumor-derived cell line. *Proc Natl Acad Sci USA* **89**: 4495–4499

Shimada H, Chatten J, Newton WA, Sachs N, Hamoudi A, Chiba T, Marsden H and Misugi K (1984) Histopathologic prognostic factors in neuroblastoma tumors: definition of subtypes of ganglioneuroblastoma and an age linked classification of neuroblastoma. *J Natl Cancer Inst* **73**: 405–416

© Cancer Research Campaign 1997

Tannock IF (1968) The relationship between cell proliferation and the vascular system in a transplanted mouse mammary tumor. *Br J Cancer* **22**: 258–273

Tannock IF (1970) Population kinetics of carcinoma cells, capillary endothelial cells, and fibroblasts in a transplanted mouse mammary tumor. *Cancer Res* **30**: 2470–2476

Thompson WD, Shiach KJ, Fraser RA, McIntosh LC and Simpson JG (1987) Tumours acquire their vasculature by vessel incorporation, not vessel ingrowth. *J Pathol* **151**: 323–332

Veis DJ, Sorenson CM, Shutter JR and Korsmeyer SJ (1993) bcl-2 deficient mice demonstrate fulminant lymphoid apoptosis, polycystic kidneys, and hypopigmented hair. *Cell* **75**: 229–240

Vogan K, Bernstein M, Leclerc JM, Brisson L, Brossard J, Brodeur GM, Pelletier J and Gros P (1993) Absence of p53 gene mutations in primary neuroblastomas. *Cancer Res* **53**: 5269–5273

Voijtesek B and Lane DP (1993) Regulation of p53 protein expression in human breast cancer cell lines. *J Cell Sci* **105**: 607–612

White AE, Livanos EM and Tisty TD (1994) Differential disruption of genomic integrity and cell cycle regulation in normal human fibroblasts by HPV oncoproteins. *Genes Dev* **8**: 666–677

White E (1994) p53, guardian of Rb. *Nature* **371**: 21–22

Wyllie AH, Kerr JFR and Currie AR (1980) Cell death: the significance of apoptosis. *Int Rev Cytol* **68**: 251–306

Yonish-Rouach E, Resnitsky D, Lotem J, Sachs L, Kimchi A and Oren M (1991) Wild-type p53 induces apoptosis of myeloid leukaemic cells that is inhibited by interleukin 6. *Nature* **352**: 345–347

Zambetti G, Bargonetti J, Walker K, Prives C and Levine AJ (1992) Wild-type p53 mediates positive regulation of gene expression through a specific DNA sequence element. *Genes Dev* **6**: 1143–1152

*© Cancer Research Campaign 1997*